# Improving Health Knowledge Through Provision of Free Digital Health Education to Rural Communities in Iringa, Tanzania: Nonrandomized Intervention Study

**DOI:** 10.2196/37666

**Published:** 2022-07-28

**Authors:** Christine Holst, Dominik Stelzle, Lien My Diep, Felix Sukums, Bernard Ngowi, Josef Noll, Andrea Sylvia Winkler

**Affiliations:** 1 Centre for Global Health Department of Community Medicine and Global Health, Institute of Health and Society University of Oslo Oslo Norway; 2 Centre for Epidemic Interventions Research Norwegian Institute of Public Health Oslo Norway; 3 Center for Global Health Department of Neurology Technical University of Munich Munich Germany; 4 Oslo Centre for Biostatistics & Epidemiology Oslo University Hospital Oslo Norway; 5 Directorate of Information and Communication Technology Muhimbili University of Health and Allied Sciences Dar es Salaam United Republic of Tanzania; 6 Muhimbili Medical Research Centre National Institute for Medical Research Dar es Salaam United Republic of Tanzania; 7 Mbeya College of Health and Allied Sciences University of Dar es Salaam Mbeya United Republic of Tanzania; 8 Basic Internet Foundation Kjeller Norway; 9 Department of Technology Systems University of Oslo Oslo Norway

**Keywords:** digital health, digital health promotion, eHealth, mobile health, mHealth, Tanzania, health education, HIV/AIDS, tuberculosis, cysticercosis, tapeworm, mobile phone

## Abstract

**Background:**

Community health education is one of the most effective measures to increase health literacy worldwide and can contribute to the achievement of specific targets of the Sustainable Development Goal 3. Digitalized health education materials can improve health knowledge as a dimension of health literacy and play an important role in disease prevention in rural sub-Saharan settings.

**Objective:**

The objective of this research is to assess the effect of a digital health education intervention on the uptake and retention of knowledge related to HIV/AIDS, tuberculosis (TB), and *Taenia solium* (neuro)cysticercosis and taeniosis in rural communities in Iringa, Tanzania.

**Methods:**

We conducted a nonrandomized intervention study of participants aged 15 to 45 years, randomly selected from 4 villages in Iringa, Tanzania. The intervention consisted of 2 parts. After the baseline assessment, we showed the participants 3 animated health videos on a tablet computer. After a period of 6 months, free access to community information spots (InfoSpots) with an integrated digital health education platform was provided to the intervention villages. Participants in the control group did not receive the intervention. The primary outcome was the difference in disease knowledge between the intervention and control groups, 12 months after baseline. Data were collected using an open-ended questionnaire, with correct or incorrect answers before and after intervention.

**Results:**

Between April and May 2019, a total of 600 participants were recruited into the intervention (n=298, 49.7%) or control (n=302, 50.3%) groups. At baseline, no statistically significant differences in knowledge of the target diseases were observed. At 12 months after intervention, knowledge about HIV/AIDS, TB, and *T. solium* (neuro)cysticercosis and taeniosis was 10.2% (95% CI 5.0%-15.4%), 12% (95% CI 7.7%-16.2%), and 31.5% (95% CI 26.8%-36.2%) higher in the intervention group than in the control group, respectively. In all 4 domains (transmission, symptoms, treatment, and prevention), an increase in knowledge was observed in all the 3 diseases, albeit to varying degrees. The results were adjusted for potential confounders, and the significance of the primary results was maintained in the sensitivity analysis to assess dropouts. The participants who reported using the InfoSpots in the 12-month assessment further increased their knowledge about the target diseases by 6.8% (HIV/AIDS), 7.5% (TB), and 13.9% higher mean proportion of correct answers compared with the participants who did not use the InfoSpots.

**Conclusions:**

Digital health education based on animated health videos and the use of free InfoSpots has significant potential to improve health knowledge, especially in rural areas of low- and middle-income countries.

**Trial Registration:**

ClinicalTrials.gov NCT03808597; https://clinicaltrials.gov/ct2/show/NCT03808597

**International Registered Report Identifier (IRRID):**

RR2-10.2196/25128

## Introduction

### Background

Health education, defined as “any combination of learning experiences designed to facilitate voluntary adaptations of behaviour conducive to health” [1], encourages behavior that promotes good health and prevents disease [2]. Health literacy is a concept defined differently across the literature but can be summarized as “an asset for improving people’s empowerment within the domains of health care, disease prevention and health promotion” [3]. Health literacy acquired through health education affects individual, family, and community health and has great potential for contributing to the achievement of specific targets of the Sustainable Development Goal (SDG; SDG 3) [4]. Health knowledge can be seen as a consequence, antecedent, or dimension of health literacy [5] and includes specific information such as prevalence, risk factors, transmission, and prevention [6]. The vast majority of people in rural communities in sub-Saharan Africa, Tanzania being the focus of this study, need health education, which is primarily provided by community health workers and individuals at health facilities, schools, and nongovernmental organizations.

The southern and eastern regions of Africa are most affected by HIV/AIDS. In 2020, 20.6 million people were living with HIV/AIDS, and 670,000 new infections and 310,000 AIDS-related deaths were registered [7]. Tuberculosis (TB) claimed 1.5 million lives worldwide in 2020 [8]. In addition, TB is the leading cause of death among HIV-infected individuals, often owing to late diagnosis, nonadherence to medication, and drug resistance. *Taenia solium* cysticercosis and taeniosis (TSCT) is considered a neglected tropical disease and is a food-borne parasitic disease that not only has a significant impact on human health by causing neurological signs or symptoms, including epileptic seizures and intestinal infection resulting in anemia and malnutrition, but also has an impact on animal health and community livelihood and can therefore be considered a one health disease par excellence [9].

The digital health landscape in Tanzania is changing in parallel with increasing mobile phone use and internet penetration. In December 2021, the country had 54.1 million mobile phone subscribers [10], with most connections in rural areas based on 2G. Coupled with significant economic growth and Tanzania’s recent shift from low-income to lower-middle–income status [11], there is great potential to reach people through their mobile phones. Adapting smart devices for health education in resource-poor areas is promising and has proven effective [12].

Few studies have addressed the development [13] and use of animated health videos (animations) to improve client knowledge in this region [14-18]. Investigating the impact of a digital health education platform in rural sub-Saharan areas with low internet connectivity is novel and forms the basis of our study.

### Objectives

The objective of the study in “the nondiscriminating access for digital inclusion” project (“DigI study”) was to assess the uptake of knowledge with regard to HIV/AIDS, TB, and TSCT and its retention over time through a digital health education intervention with animations and provision of free access to community information spots (InfoSpots) with an integrated digital health education platform.

## Methods

### Study Design and Setting

As published in the DigI study protocol [19], this was a nonrandomized intervention study formatted as a community-based and longitudinal study. The study was undertaken in 4 villages with their 21 subvillages in the Iringa region located in the southern highlands of mainland Tanzania. The Iringa region is endemic to HIV/AIDS [20], TB [21], and TSCT [22], making prevention strategies for the target diseases essential. The Iringa district is a rural area in the northern Iringa region, where agriculture and livestock are the main sources of income [23]. All the 4 villages included in this study had health facilities.

**Figure 1:**
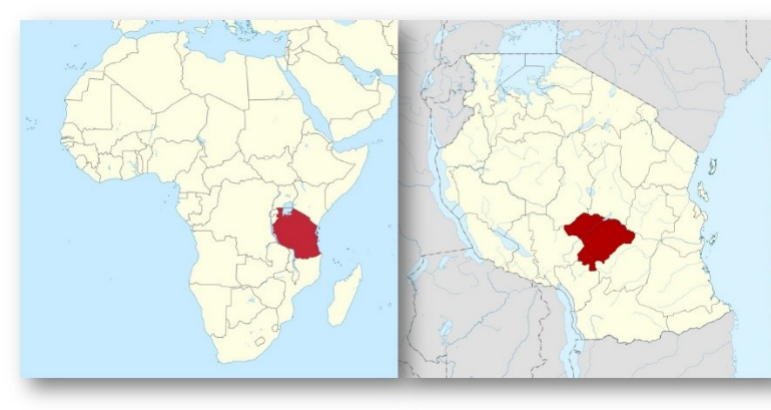
Geographic location of the study site. From left to right: Tanzania within Africa and the Iringa district within Tanzania. Both illustrations are from Wikimedia Commons contributors (CC BY-SA 3.0).

Households in Migoli and Izazi (Figure 1) became the intervention group, whereas households in Kimande and Idodi formed the control group. The intervention and control villages were purposively chosen based on technical installation reasons and a traveling distance of >2.5 hours by car between the intervention and control villages.

### Participants

In April and May 2019, a total of 991 households were assessed for their eligibility to participate in the study. People aged between 15 and 45 years living permanently in randomly selected households from all subvillages were eligible for inclusion in the study. People who planned to move to areas outside the villages during the data collection period were not eligible to participate. A female or male member per household was randomly selected using the Kish-grid method [24] to participate in the study based on multilevel sampling.

### Intervention

**Figure 2:**
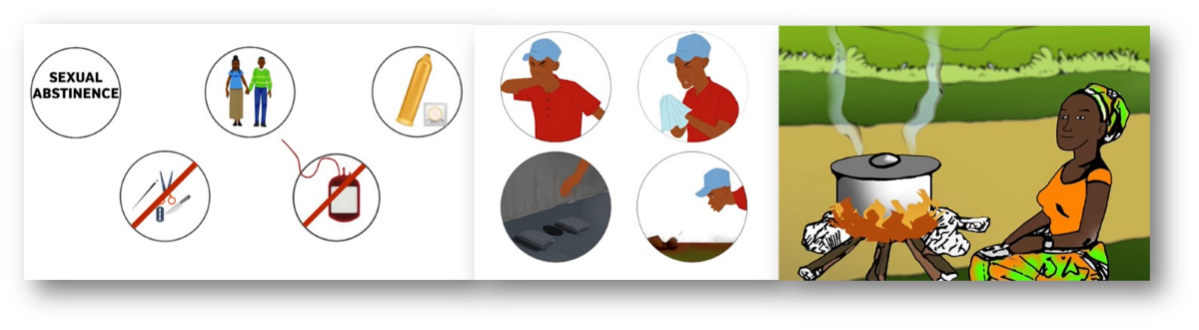
Examples of illustrations of key messages from the animations. Important key messages were, for example, “never share sharp objects” and “avoid unsafe blood transfusions” for HIV/AIDS, “cover mouth and nose with paper or cloth when coughing or sneezing” for tuberculosis, and “cook meat well” for Taenia solium cysticercosis and taeniosis. These key messages were well illustrated, narrated, and repeated in the animations.

The DigI study intervention consists of 2 parts: animations with key messages on HIV/AIDS, TB, and TSCT and the digital health education platform. The first part of the intervention—3 stories told with animations about the target diseases (Figure 2)—was presented to participants by Tanzanian members of the study team. The animations were shown on a tablet to the participants privately in their homes after the baseline assessment using the questionnaire. The animations, lasting between 3 and 7 minutes, included key messages for 4 domains (transmission, symptoms, treatment, and prevention) in each disease (HIV/AIDS, TB, and TSCT).

The DigI team applied an interdisciplinary approach when creating digital health messages based on a process involving local stakeholders and government-approved health promotion materials in a nondigital format [25]. All the animations were bilingual in Swahili and English [26]. All participants in the intervention group viewed animations in the Tanzanian Swahili language.

**Figure 3:**
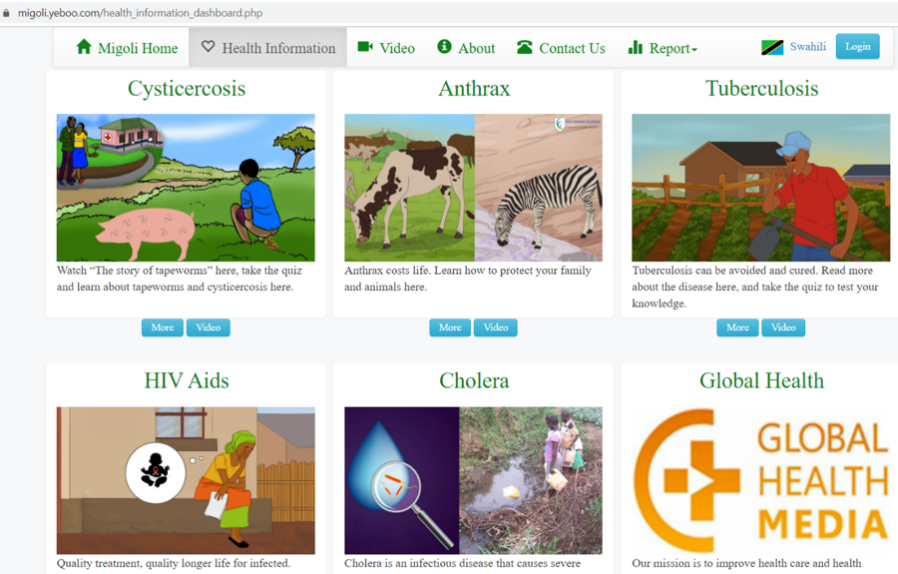
Screenshot of the platform.

The second, unsupervised and voluntary part of the intervention started 6 months after the baseline assessment, with the rollout of the digital health education platform (the platform) in the already established InfoSpots in the intervention villages. The platform [27] could be accessed openly and free of charge via the participants’ own smartphones or via public tablets provided at the InfoSpots. In addition to the animations in the first part of the intervention, the platform contained additional text information, graphics, and quizzes. We installed 5 InfoSpots in Migoli and Izazi, covering village offices and health facilities as well as Nyerere High School in Migoli. The animations (English versions) and a screenshot of the platform can be found in Figure 3 and Multimedia Appendices 1-3.

The control villages did not receive any intervention. Communities in the region usually access general information, health information, and education and communication materials through various channels. These channels include radio, television, newspapers, community meetings, and outreach from schools via children as well as when visiting health facilities.

### Questionnaire

An open-ended Swahili-based questionnaire was used for data collection. The questionnaire was designed by Tanzanian and international experts in English (Multimedia Appendix 4), based on questions from previous studies [28,29] (The International Organisation for Migration, unpublished data, January 2014) and expanded through additional questions derived from approved health information provided by the Tanzanian Ministry of Health. The questions were translated to Swahili and back-translated into English again. A pilot study (in Swahili) was conducted with 50 participants before data collection [19]. A total of 7 questions were adapted to increase the participants’ comprehensibility. The questionnaire contained questions that could have more than one correct answer. Answers to the questions were binary (correct or incorrect. The questions were asked face to face and the enumerator entered the participant’s answers into a digital form. To analyze the health knowledge of the participants, we used 12 HIV/AIDS-knowledge questions, with 23 possible correct answers, 8 TB questions with 30 possible correct answers, and 13 TSCT questions with 22 possible correct answers. The overall Cronbach α, per disease, ranged between .79 and .87.

### Outcomes

The primary outcome of the DigI study was the difference in the overall proportion of correct answers per target disease between the intervention and control groups 12 months after baseline assessment. The main hypothesis was that the proportion of correct answers in the intervention group would differ from the proportion of correct answers in the control group at 3 and 12 months. The secondary outcome was the difference in the proportion of correct answers in the 4 domains of each disease: transmission, symptoms, treatment, and prevention. The secondary hypothesis was that there would be differences among the groups’ proportion of correct answers per domain. For all outcome variables, the questionnaire and corresponding correct responses were used.

To investigate the effect of the digital health education intervention, we assessed the disease knowledge of both the intervention and control groups at baseline and at 3 and 12 months after the first part of the intervention. Participants in the intervention group were further asked questions related to their views regarding animations at the 3-month follow-up and their use of the InfoSpots at the 12-month follow-up.

### Statistical Methods

For the sample size calculation, we assumed a proportion of 50% of correct answers in the group without intervention and a difference among the groups of 15% to 20% after the intervention. The power was set to 80% and a 2-sided significance level of 5% (using Bonferroni correction) was applied. The target sample size was 460. To increase the precision of the key estimates and account for dropouts, 600 participants were recruited.

Sociodemographic sample characteristics were described by frequency with percentage or mean with SD. Differences in sociodemographic characteristics among the groups were tested using Pearson chi-square test (for categorical variables) and a 2-independent sample 2-tailed *t* test (for age). For each disease, the total number of correct answers was summed for each participant and divided by the maximum number of correct answers to generate the overall proportion of correct answers. The same calculations were performed to generate the proportion of correct answers for each domain per disease. Assuming missing data at random, a mixed effects linear regression model for continuous outcomes was used to examine the difference in the average proportion of correct answers between the intervention and control groups. The analysis was performed with and without adjusting for age, sex, education, and occupation as potential confounders that could have influenced the outcome. In the mixed model of linear regression, the subvillage, household, and time point were specified as random variables and the group, as a fixed variable. Unstructured and exchangeable variance-covariance structures for the households and time were chosen. To assess the impact of time with and without adjusting, an interaction term “group×time” was added to the mixed model. The results of the analysis using mixed effects linear regression were reported as the average difference among the groups, and 95% CI were calculated. The *P* value was also reported for additional information. Loss to follow-up was addressed by sensitivity analysis assuming a worst-case scenario, with all answers being incorrect after the dropout of the participant. The differences in the proportion of correct answers between the participants who used the InfoSpots and those who did not were described using mean and SD. Analyses were performed using Stata/SE (version 16; StataCorp) [30].

### Ethics Approval

The Tanzanian National Health Research Ethics Sub-Committee granted ethical clearance (NIMR/HQ/R.8a/Vol. IX/2947) in November 2018 and extended its clearance in February 2020. The Tanzania Commission for Science and Technology issued a research permit in March 2019. The DigI study was registered at ClinicalTrials.gov (NCT03808597) before enrolling the first participant. Each participant was informed orally and in writing in Swahili. Informed consent to participate in the study was obtained in writing, and parental consent was required for participants aged <18 years.

## Results

### Study Population

**Figure 4:**
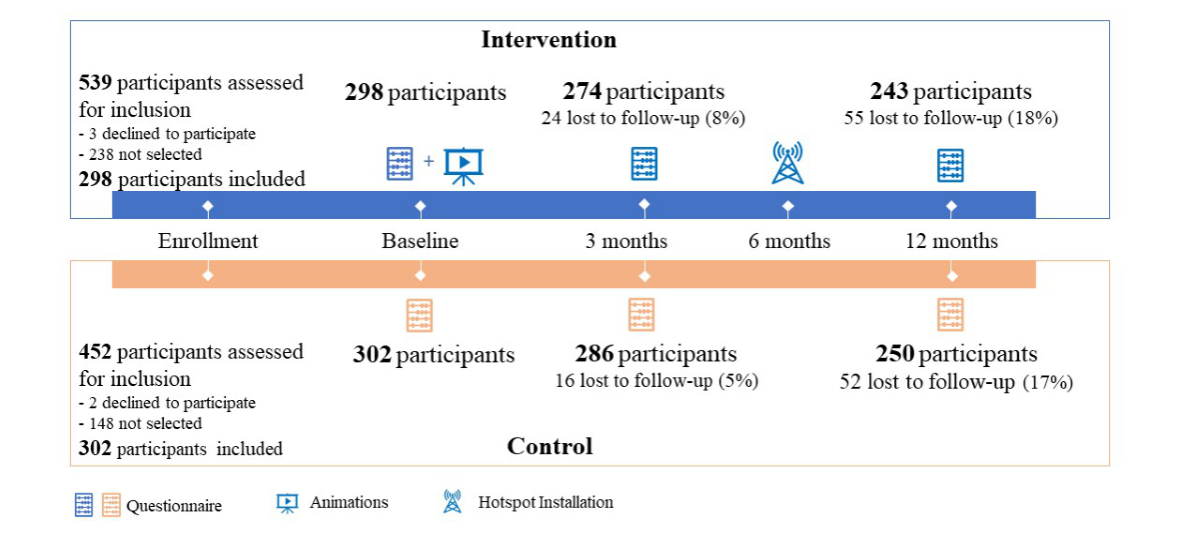
Flowchart of the study. The figure shows the number of households assessed for inclusion before a random generator selected the 298 households for the intervention group and 302 households for the control group. One participant per household was included in the study. Further, the figure shows the remaining participants at the 3- and 12-month assessment.

A total of 600 participants were recruited into the study, of whom 560 (93.3%) were interviewed at 3-month follow-up and 493 (82.2%) at 12-month follow-up (see Figure 4 for a flowchart of the study).

The descriptions of the 2 study populations are presented in Table 1. The main differences were in education and occupation, which we adjusted for in our analysis.

**Table 1:** Baseline demographic characteristics of participants aged 15 to 45 years living in the intervention and control villages.

Characteristics			Total (N=600)		Intervention (n=298)		Control (n=302)
**Sex, n (%)**							
	Female	362 (60.3)		193 (64.8)		169 (56)	
	Male	238 (39.7)		105 (35.2)		133 (44)	
Age (years), mean (SD)			30.0 (8.3)		28.7 (8.1)		30.8 (8.3)
**Village, n (%)**							
	Migoli	173 (28.8)		173 (58.1)		N/A^a^	
	Izazi	125 (20.8)		125 (41.9)		N/A	
	Idodi	150 (25)		N/A^a^		150 (49.7)	
	Kimande	152 (25.3)		N/A		152 (50.3)	
**Education, n (%)**							
	No education	79 (13.2)		56 (18.8)		23 (7.6)	
	Primary School	427 (71.2)		181 (60.7)		246 (81.5)	
	Secondary or higher education	94 (15.7)		61 (20.4)		33 (11)	
**Occupation, n (%)**							
	Petty traders	151 (25.2)		135 (45.3)		16 (5.3)	
	Farmers	321 (53.5)		65 (21.8)		256 (84.8)	
	Other	128 (21.3)		98 (32.9)		17 (9.9)	

^a^N/A: not applicable.

### Knowledge Outcomes

**Figure 5:**
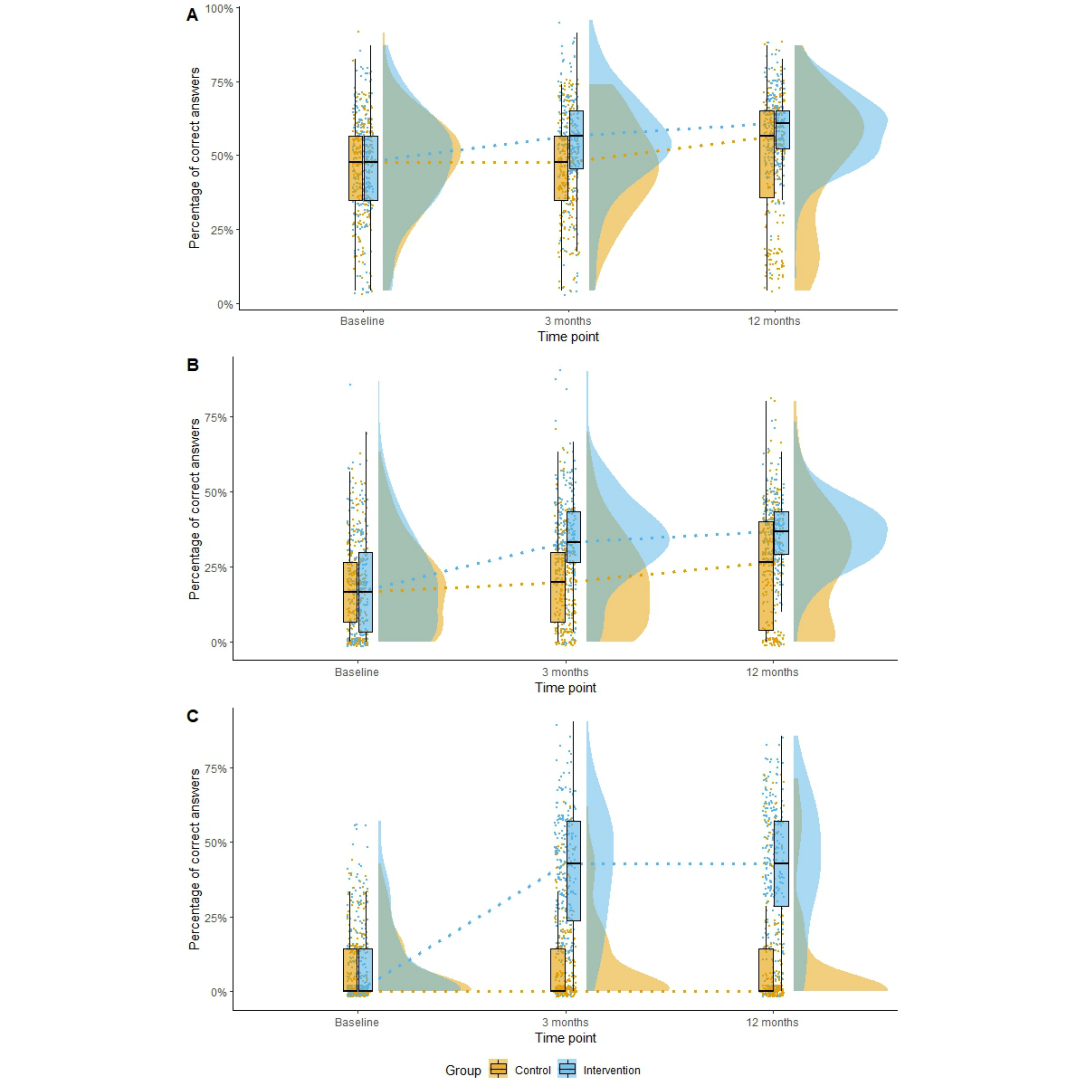
Box plot and density plots for knowledge and by disease, group, and time point: (A) HIV/AIDS, (B) tuberculosis, (C) Taenia solium cysticercosis and taeniosis.

At baseline, all (600/600, 100%) participants reported that they had heard of HIV/AIDS. Overall, 91% (546/600) of the participants had heard of TB, but only 9% (54/600) of the participants had heard of cysticercosis, and 13.2% (79/600) of the participants had heard of the pork tapeworm. Overall, only 3% (18/600) of the participants said that they knew what pork with cysticercosis looked like. The baseline knowledge, calculated as the unadjusted mean percentage of correct answers, was similar in both groups, although it was slightly higher for TB and TSCT in the intervention group than in the control group. The overall proportions of correct answers at the disease level are presented in Table 1 and Figure 5. The equivalent responses broken down at the domain level are presented in Table 2.

At the 3-month assessment, correct answers regarding HIV/AIDS in the intervention group increased to a mean percentage of 54.7% (SD 17%), whereas they remained at 47.1% (SD 16.6%) in the control group (adjusted difference 8.3, 95% CI 3.2-13.4). The intervention effect was larger in the TB section, with an increase from baseline of 15.5% to 34.4% (SD 15.4%) in the intervention group compared with a 1.5% increase in the control group (adjusted difference 15.8, 95% CI 11.7-20.0). For TSCT, the first part of the intervention led to a 31.3% knowledge increase, reaching 41% (SD 20.2%) in the intervention group, whereas only a limited increase of 2.5% was measured in the control group (adjusted difference 31.2, 95% CI 26.9-35.6) after 3 months.

At the 3-month assessment, 97.1% (266/274) of the participants in the intervention group reported learning through animations, and 74.1% (203/274) reported having discussed health messages with friends and family.

**Table 2:** Overall intervention effect for knowledge about the target diseases and mean percentage differences among the groups (N=600).

Target disease and time point			Correct answers, mean (SD)				Crude difference, mean (95% CI)		Adjusted difference, mean (95% CI)		*P* value (adjusted model)	
			Intervention		Control							
**Overall HIV/AIDS**												<.001
	Baseline	47.6 (17.4)		48.3 (15.6)		−0.1 (−6.5 to 6.3)		0.3 (−4.7 to 5.4)				
	3 months	54.7 (17.0)		47.1 (16.6)		8.0 (1.6 to 14.4)		8.3 (3.2 to 13.4)				
	12 months	59.8 (11.9)		50.2 (21.1)		9.9 (3.4 to 16.3)		10.2 (5.0 to 15.4)				
**Overall tuberculosis**												<.001
	Baseline	18.9 (16.2)		18.3 (14.7)		1.3 (−3.6 to 6.1)		2.0 (−2.1 to 6.1)				
	3 months	34.4 (15.4)		19.8 (15.0)		15.2 (10.3 to 20.0)		15.8 (11.7 to 20.0)				
	12 months	35.8 (11.5)		25.1 (18.5)		11.3 (6.3 to 16.2)		12.0 (7.7 to 16.2)				
**Overall** *Taenia solium* **cysticercosis and taeniosis**												<.001
	Baseline	9.7 (14.9)		7.6 (12.1)		2.4 (−1.7 to 6.4)		2.6 (−1.6 to 6.8)				
	3 months	41.0 (20.2)		10.1 (15.1)		31.1 (26.9 to 35.3)		31.2 (26.9 to 35.6)				
	12 months	42.1 (22.0)		11.0 (20.0)		31.4 (26.8 to 35.9)		31.5 (26.8 to 36.2)				

Participants in the intervention group were able to maintain their knowledge of the disease, as documented at the 12-month assessment. Participants in the control group were found to have a slight increase in knowledge of 1.9% (HIV/AIDS), 6.8% (TB), and 3.4% (TSCT) from baseline, which was significantly lower than the increase of 12.2% for HIV/AIDS (adjusted difference 10.2, 95% CI 5.0-15.4), 16.9% for TB (adjusted difference 12.0, 95% CI 7.7-16.2), and 32.4% for TSCT (adjusted difference 31.5, 95% CI 26.8-36.2) in the intervention group. After 12 months, participants in the intervention group retained a mean proportion of correct answers of 59.8% for HIV/AIDS, 35.8% for TB, and 42.1% for TSCT.

The intervention effect was statistically significant in the crude analyses and remained statistically significant after adjusting for age, sex, education, and occupation. Figure 5 shows the corresponding raincloud plot illustrating the median percentage of correct answers in both groups at all time points.

The intervention effect in the transmission HIV/AIDS domain was statistically significant, with an adjusted mean difference of 6% (95% CI 1.7%-10.4%) after 3 months and 6.4% (95% CI 1.9%-10.9%) after 12 months. In this domain, the effect remained unchanged over time (*P*=.06), in contrast to the other domains, where the effect either increased or decreased between 3 and 12 months. The effect was significant for all TB domains, although knowledge increase at 12 months was reduced compared with that at 3 months. However, as with the overall scores, the strongest effect was observed for TSCT, for which there were low baseline levels of disease knowledge. The adjusted mean percentage difference between the 2 groups reached 40% in 3 out of the 4 domains after 3 months and in 2 out of the 4 domains after 12 months. See Table 3 for all domain level values.

A sensitivity analysis assessing the dropout of 107 participants (n=55, 51% in the intervention group and n=52, 49% in the control group) and assuming a worst-case scenario showed an unchanged effect for HIV/AIDS, a 3% reduction in the intervention effect for TB, and a 5.8% reduction in the intervention effect for TSCT.

At the 12-month assessment, 30% (73/243) of the participants in the intervention group reported having accessed the InfoSpots with the health education platform. Of these, 53% (39/73) had viewed HIV/AIDS animation, 37% (27/73) had TB animation, and 44% (32/73) had TSCT animation. Those who visited the InfoSpots further increased their knowledge by 8.5% (HIV/AIDS), 6.3% (TB), and 12.1% (TSCT), whereas participants who did not visit the InfoSpots remained at the same knowledge level (Table 4).

**Table 3:** Intervention effect and mean percentage differences among the groups at the domain level.

Domains and time point				Correct answers, mean (SD)			Crude difference, mean (95% CI)	Adjusted difference, mean (95% CI)		*P* value (adjusted model)	
				Intervention	Control						
**HIV/AIDS**											
	**Transmission**										.06
		Baseline	49.5 (20.0)		47.8 (18.7)	2.0 (−4.0 to 7.9)			1.9 (−2.3 to 6.2)		
		3 months	54.0 (19.4)		47.8 (20.2)	6.2 (0.2 to 12.1)			6.0 (1.7 to 10.4)		
		12 months	59.2 (16.8)		52.5 (21.3)	6.4 (0.4 to 12.5)			6.4 (1.9 to 10.9)		
	**Symptoms**										<.001
		Baseline	79.2 (33.4)		81.1 (30.9)	−1.4 (−11.5 to 8.7)			−2.2 (−11.1 to 6.8)		
		3 months	88.0 (28.3)		71.8 (37.1)	16.5 (6.4 to 26.7)			15.5 (6.5 to 24.6)		
		12 months	93.6 (19.0)		75.0 (40.6)	18.8 (8.5 to 29.1)			17.9 (8.6 to 27.1)		
	**Treatment**										<.001
		Baseline	70.5 (29.5)		73.7 (27.6)	−2.4 (−12.6 to 7.8)			−2.8 (−11.9 to 6.3)		
		3 months	77.2 (25.1)		67.3 (30.5)	10.6 (0.4 to 20.8)			10.1 (1.0 to 19.2)		
		12 months	90.7 (18.1)		72.8 (37.2)	18.5 (8.1 to 28.8)			18.1 (8.8 to 27.3)		
	**Prevention**										<.001
		Baseline	35.3 (19.2)		36.8 (17.7)	−0.8 (−6.6 to 5.1)			0.2 (−4.7 to 5.1)		
		3 months	43.9 (19.1)		37.5 (16.3)	7.0 (1.2 to 12.8)			7.9 (2.9 to 12.8)		
		12 months	46.8 (15.6)		39.0 (19.9)	8.4 (2.5 to 14.2)			9.2 (4.2 to 14.3)		
**Tuberculosis**											
	**Transmission**										<.001
		Baseline	17.6 (22.9)		17.0 (17.3)	1.6 (−2.9 to 6.1)			2.1 (−1.7 to 6.0)		
		3 months	29.6 (17.5)		18.8 (14.5)	11.5 (7.3 to 15.7)			12.1 (8.5 to 15.6)		
		12 months	28.7 (14)		21.1 (16.2)	8.5 (4.2 to 12.7)			9.0 (5.3 to 12.6)		
	**Symptoms**										<.001
		Baseline	14.4 (15.5)		13.8 (13.4)	1.3 (−3.2 to 5.7)			1.8 (−2.1 to 5.6)		
		3 months	28.7 (18.1)		15.2 (14.5)	14.0 (9.5 to 18.5)			14.4 (10.5 to 18.4)		
		12 months	28.1 (15.0)		19.9 (18.4)	8.6 (4.0 to 13.3)			9.1 (5.0 to 13.2)		
	**Treatment**										<.001
		Baseline	54.8 (38.3)		53.8 (37.4)	2.3 (−9.0 to 13.5)			4.5 (−6.1 to 15.0)		
		3 months	82.2 (28.6)		53.6 (38.4)	29.5 (18.5 to 40.5)			31.6 (21.2 to 41.9)		
		12 months	89.0 (20.7)		61.6 (40.7)	28.4 (17.2 to 39.6)			30.4 (19.9 to 41.0)		
	**Prevention**										<.001
		Baseline	13.6 (18.5)		13.5 (16.9)	0.5 (−4.1 to 5.1)			1.1 (−3.0 to 5.2)		
		3 months	29.2 (16.3)		15.3 (16.2)	14.1 (9.6 to 18.7)			14.6 (10.5 to 18.7)		
		12 months	33.2 (13.4)		22.5 (19.8)	10.9 (6.3 to 15.6)			11.4 (7.3 to 15.6)		
*Taenia solium* **cysticercosis and taeniosis**											
	**Transmission**										<.001
		Baseline	8.7 (19.4)		4.7 (14.1)	4.2 (−1.3 to 9.7)			4.1 (−1.5 to 9.7)		
		3 months	50.1 (28.7)		8.3 (19.1)	42.0 (36.2 to 47.7)			41.7 (35.9 to 47.5)		
		12 months	51.4 (27.0)		10.0 (23.4)	41.5 (35.4 to 47.6)			41.2 (35.0 to 47.3)		
	**Symptoms**										<.001
		Baseline	6.0 (9.9)		5.6 (8.8)	0.9 (−1.8 to 3.7)			1.0 (−1.7 to 3.8)		
		3 months	22.5 (19.9)		7.6 (10.6)	15.3 (12.4 to 18.3)			15.4 (12.4 to 18.5)		
		12 months	27.1 (22.2)		8.1 (15.4)	19.5 (16.2 to 22.8)			19.6 (16.1 to 23.1)		
	**Treatment**										<.001
		Baseline	7.4 (26.2)		5.0 (21.8)	2.4 (−3.3 to 8.0)			2.8 (−3.5 to 9.2)		
		3 months	65.6 (47.6)		16.2 (36.9)	49.3 (43.2 to 55.7)			49.5 (42.5 to 56.5)		
		12 months	72.8 (44.6)		26.0 (44.0)	46.5 (39.0 to 53.9)			46.7 (38.7 to 54.6)		
	**Prevention**										<.001
		Baseline	16.6 (24.0)		14.0 (22.4)	2.5 (−3.3 to 8.3)			2.8 (−2.9 to 8.5)		
		3 months	55.4 (22.7)		14.6 (21.6)	40.7 (34.8 to 46.5)			40.8 (35.1 to 46.6)		
		12 months	50.1 (26.4)		13.7 (25.5)	36.3 (30.2 to 42.5)			36.5 (30.5 to 42.6)		

**Table 4:** Comparison of knowledge about the 3 target diseases, shown as mean percentage of correct answers, between participants who used the information spots (InfoSpots) and those who did not at the 12-month assessment.

Target disease and time period			InfoSpots users (n=73), mean (SD)		Non–InfoSpots users (n=170), mean (SD)
**HIV/AIDS**					
	3 months	55.1 (18.0)		53.1 (16.0)	
	12 months	63.6 (11.1)		56.8 (11.6)	
**Tuberculosis**					
	3 months	34.8 (16.5)		33.9 (15.1)	
	12 months	41.1 (11.4)		33.6 (10.8)	
*Taenia solium* **cysticercosis and taeniosis**					
	3 months	38.5 (20.3)		39.9 (20.3)	
	12 months	50.6 (17.6)		36.7 (21.8)	

## Discussion

### Principal Findings

The DigI study is the first of its kind to assess health knowledge uptake and retention following a 2-pronged digital health education intervention consisting of watching health animations and accessing a free digital health education platform (InfoSpots) in rural Tanzania.

Participants in the intervention group attained significantly higher levels of knowledge than those in the control group, with the adjusted differences among the groups being 10.2%, 12%, and 31.5% for HIV/AIDS, TB, and TSCT, respectively, after 12 months. We observed increased knowledge of all 3 diseases in all domains. The effect on knowledge retention was particularly strong for TSCT, with a low level of knowledge at baseline, as shown by the increase from 9.7% to 42.1% after 12 months.

Looking at the domains, the effect was strongest in the 3-month assessment for all TB and 3 out of the 4 TSCT domains. In contrast, for all HIV/AIDS and TSCT symptoms domains, an increased effect was observed at the 12-month assessment, indicating that the use of InfoSpots for health education may have contributed to the retention and enhancement of health knowledge. In fact, the InfoSpots users showed further increase in knowledge at 12 months compared with those who did not use InfoSpots. This increase in knowledge was more pronounced for HIV/AIDS and TSCT than for TB. However, this does not exclude the possibility that the participants accessed other knowledge sources over the course of the study. This may also explain the slight increase in knowledge in the control group at 12 months, which was 1.9%, 6.8%, and 4.4% for HIV/AIDS, TB, and TSCT, respectively. We were unable to assess why the participants in the control group improved their knowledge. They were not asked any questions about whether they had talked to other villagers about diseases or had otherwise obtained information. Contamination among the groups is unlikely, given the long distance of at least two and a half hours by car. Between the baseline and the 12-month assessment, 107 out of 600 participants dropped out, accounting for 17.8% of the initial participants. All participants were contacted by subvillage leaders via their mobile phone number. The reasons for dropping out were, in most cases, migration for work opportunities in other villages, as the data collection lasted for more than a year in an area characterized by seasonal workers in agriculture, fishing activities, and petty trading.

The intervention effect was independent of age, sex, education, and occupation and demonstrated that digital health education is applicable and useful for all people despite sociodemographic differences.

### Comparison With Prior Work

The DigI study shows that animations with short, clear, and basic health messages have significant potential to improve health knowledge in the short to long term, as confirmed by previous studies in a sub-Saharan setting [14-18]. Despite challenges in comparing our results directly with other intervention studies using animations for health knowledge transfer—owing to differences in health promotion content, interventions, target groups, and research design, 3 studies are worth mentioning. Adam et al [18] observed a small significant increase in knowledge among pregnant women in a month, but not 5 months, after their video breastfeeding intervention. Lund et al [16] found a 38% increase in maternal health knowledge among health care workers in their intervention study 12 months after introducing a safe delivery app with animations for health knowledge improvement, whereas Bolan et al [17] researched the same app and found a 17% to 19.5% increase in their 3-month assessment. Although health care workers may have some prior medical knowledge, the results indicate that the study population and setting, and most probably other factors, may impact knowledge uptake and retention and always need to be considered.

### Strengths and Limitations

Overall, the DigI study, which offers digital health education both directly and via local InfoSpots, is unique and novel and could inform the rethinking of health education delivery in rural areas of low- and middle-income countries as a whole. It seems that simple animations co-designed with local stakeholders, provided in Swahili with local characters in a recognizable environment [25], can contribute to improvement in health knowledge. The repetition of key messages in animations and illustrative scenes accompanied by the narrator’s explanations (Figure 2) could be key factors for better knowledge uptake and retention.

Community health workers, equipped with a digital tool, could share tailored health messages while simultaneously sensitizing individuals to the use of a locally adapted digital health promotion platform that can be deployed at strategic locations within the communities. In fact, we were able to show that InfoSpots users were creative and enthusiastic when using the platform and that the animations were well accepted as a knowledge transfer tool in the communities [31]. Although InfoSpots require cross-sectoral collaboration among community leaders, internet providers, and the health and educational sectors, if co-designed and promoted together, they have the potential to create ownership and thus achieve long-term sustainability. Furthermore, such InfoSpots can contribute to the development of digital skills and provide people with access to essential information, thus supporting the achievement of SDG 3, SDG 4, and SDG 16.

The DigI study has some limitations that require further investigation. First, the selection of intervention and control villages was nonrandomized and performed for technical reasons related to the installation of the InfoSpots. The demographic data showed differences between the intervention and control groups in terms of age, sex, education, and occupation. This may have affected the external validity of the DigI study’s research design. However, our adjusted results showed that none of the possible confounding factors affected the intervention effect. Participants were randomly selected from all villages and subvillages, which is a strength that reduces the selection bias. Furthermore, with this study design, it was not possible to assess whether improved health knowledge and resulting health literacy may have an impact on the burden of disease from our 3 target diseases in the communities. Increased health knowledge does not guarantee a change in health behaviors. To initiate and sustain behavior change, multiple interventions at various levels are required [1], and this project only focused on interventions that improve health knowledge.

### Future Directions

In summary, the DigI study showed that the unique combination of animated health videos shown to participants in their homes and the use of free InfoSpots with an integrated digital health platform improved knowledge about the 3 target diseases, not only in the short term but also over a longer time frame. Furthermore, implementing health access points such as InfoSpots in rural Tanzania, with animations and other digital health education materials in their own language, may contribute to achieving SDG 3 and in our case, particularly target SDG 3.3—ending the epidemics of HIV/AIDS, TB, and neglected tropical diseases by 2030. However, further studies are required to demonstrate the impact of the promising digital health intervention of the DigI study on health-seeking behavior and thus on disease prevention and management.

## Appendix

Multimedia Appendix 1 HIV/AIDS health animated video (English).

Multimedia Appendix 2 Tuberculosis health animated video (English).

Multimedia Appendix 3 Taenia solium cysticercosis and taeniosis health animated video (English).

Multimedia Appendix 4 Study questionnaire (HIV, tuberculosis, or Taenia solium cysticercosis and taeniosis knowledge questions used at baseline and at 3 and 12 months).

## Abbreviations

InfoSpots: information spots
SDG: Sustainable Development Goal
TB: tuberculosis
TSCT: Taenia solium cysticercosis and taeniosis

## Data Availability

Deidentified participant-level data and the data dictionary will be made available on request, with a signed data access agreement after December 2022. Please email the corresponding author for their access.
